
JOURNAL INFORMATION
==============================
NLM Title Abbreviation: J Can Health Libr Assoc
Journal ID: J Can Health Libr Assoc
NLM Journal ID: 101229936
Journal ID: JCHLA

The Journal of the Canadian Health Libraries Association

EISSN: 1708-6892
Publisher: Canadian Health Libraries Association / Association des bibliotèques de la santé du Canada

ARTICLE INFORMATION
==============================
PMCID: PMC10403117
DOI: 10.29173/jchla29721
Article ID: JCHLA-44-064
Article version: 1
Subjects: Abstracts

CHLA 2023 CONFERENCE LIGHTNING TALKS / ABSC CONGRÈS 2023 PRÉSENTATIONS ÉCLAIR

Electronic publication date: 2023 Aug 1
Publication date: 2023 Aug
Volume: 44
Issue: 2
First page: 64
Last page: 69
License: This article is distributed under a Creative Commons Attribution License: https://creativecommons.org/licenses/by/4.0/
License URL: https://creativecommons.org/licenses/by/4.0/

==============================

## LT1. Factoring into impact: Librarian involvement on knowledge synthesis projects and relationship to journal impact factor

Krista Alexander, Katharine Hall
Concordia University

**Introduction:** There are numerous benefits for researchers to involve a librarian in their knowledge synthesis projects such as better search precision and higher quality search strategy and methodological reporting. However, does librarian involvement make a difference when it comes to publication venue? This research examines if any correlation exists between librarian involvement on published knowledge syntheses, and the impact factor of the journals where they are published. **Methods:** Focusing on the journals from a single category ('Psychology, Clinical') in Clarivate's Journal Citation Reports (JCR), the authors analyzed the librarian involvement (co-author, acknowledged, unclear or none) in a complete set of English language knowledge syntheses published over a one-year period (2020). **Results:** Librarians were co-authors on 2.7%, and acknowledged in 11.7% of the 551 knowledge syntheses examined. Dividing the included documents into similar sized groups, we defined four impact factor ranges: G1=12.792 to 7.169; G2=6.724 to 4.507; G3=4.473 to 3.368; G4=3.311 to 0.342. The proportion of knowledge syntheses with librarian co-authors and librarian acknowledgements were, respectively; G1: 3% and 11.1%; G2: 2.1% and 7.7%; G3: 5.1% and 16.7%; and G4: 0.7% and 11.9%. **Discussion:** Although G3 showed higher percentages of librarian co-authorship and acknowledgment than the other groups, the number of librarian co-authored papers in the document set was too low for chi-squared significance testing. Further research in a JCR category with a higher proportion of librarian co-authorship may be necessary to determine if there is a relationship between journal impact factor and librarian involvement.

## LT2. Diversity audits in health library collections: a methodological exploration

Nicole Askin
University of Manitoba

**Introduction:** Despite the increasing popularity of diversity audits in public, school and academic libraries, there are limited examples of comprehensive audits performed in the context of a health library. Methodological approaches from the literature require adaptation to meet this unique context. **Methods:** Peer-reviewed and grey literature was reviewed to compile described methods of assessing collections with respect to diversity, equity, inclusion, and accessibility (DEIA). Work was then done to operationalize how these methods could be applied in health. **Results:** Seven categories of assessment were identified: ● Diversity of the producer: who is the creator of the work and who publishes it. ● Diversity of the content: what topics are covered and is the terminology inclusive. ● Diversity of appearance: what is shown on the cover and are the images within representative. ● Findability/indexing of the resource: use of appropriate and respectful metadata. ● Accessibility of the resources: availability of alternative formats and languages, accessibility features. ● Comparisons to established lists, such as bibliographies or award winners. There were also different approaches to defining 'diversity' in the context of a diversity audit, with as many as twenty possible facets to what could be counted. **Discussion:**

Because many of the existing diversity audit examples are from the public/school library context, there are challenges in operationalizing their methods to health libraries. Further research is needed particularly around the question of appropriate targets for representation of diversity in the health collection. This project represents a starting point for discussion around comprehensive diversity auditing in health libraries.

## LT3. The challenge of accurately formatted references: Does Medline get it right?

Sunny Chung, Laurel Scheinfeld
Stony Brook University

**Introduction:** Health sciences libraries in both academic and clinical contexts are becoming more involved in furthering their institutions' research mandates, and librarians are increasingly called upon to support their patrons' research needs. A literature scan revealed many libraries are writing about their research support initiatives and the changing roles of librarians, but no syntheses exist on library research support services in health sciences contexts. We conducted a scoping review to map the academic literature on this topic and to assist libraries as they continue to adapt to researchers' evolving needs. **Methods:** We searched Medline, Embase, ERIC, LISTA, LISS, Scopus and Web of Science to identify reports of research support services provided in health sciences libraries. An advanced Google search for grey literature was also conducted. Articles describing development, implementation, or evaluation of research support initiatives provided in a health sciences library were included. Data extraction will focus on library context, user characteristics, and services implemented. Findings will be summarized using evidence maps in order to facilitate knowledge translation. **Results:** The database searches returned 6336 results, and after removing duplicates, 3545 records remained for title/abstract screening. Full-text screening was conducted on 277 articles. Preliminary results show a predominance for systematic review support and the emergence of data management support. **Discussion:** This scoping review maps out the existing literature on research support practices in the health sciences context. It provides health sciences libraries with a benchmark from which they can assess and develop their own research services.

## LT4. Charting Our Sustainable Future: Librarian Philosophies to Discover Meaning in What We Do

Justin Fuhr
University of Manitoba

**Introduction:** Similar to a teaching philosophy, a librarian philosophy is a reflection of a librarian's approach to the profession. A librarian philosophy summarizes a librarian's professional practice and their standards and values, based on personal and professional beliefs. **Description:** In 2020, I wrote my librarian philosophy based on personal and professional experiences. I reflected on what is meaningful to me and how I approach my work. I came up with four areas of emphasis: community building, meaningful work, lifelong learning, and supporting others. **Outcomes:** As an early-career librarian, I want to ground my decision-making early in my career. With my librarian philosophy, I frame current and future action on my personal beliefs and standards, giving me increased confidence. Having a document based on my values allows day-to-day values-based decision-making. My librarian philosophy is posted publicly on my University of Manitoba Libraries' profile, and has guided me in my decision-making and goal setting. Students and faculty can publicly view how I approach librarianship; this gives transparency to how I provide library services for my clients. Librarian philosophies can also provide direction to your career and prevent burn out by intentionally infusing meaning throughout your work. **Discussion:** Reflection allows librarians to change their practice based on experience. In doing so, librarians clarify the work they do and why they do it. This guides future action and leads to a meaningful career. In developing a personal librarian philosophy, you chart a sustainable path, one that aligns with your values, and you can be an active participant in intentional and engaged librarianship.

### LT5. Building Critical Health Literacy Capacity in Partnership with Communities: Research in Progress

Vera Granikov
Centre de recherche du CHUM

**Introduction:** The Covid-19 pandemic has highlighted the overwhelming complexity of health information and the importance of critical health literacy. One way to improve critical health literacy at the community level is through collaborative learning, where group members share and question their experiences and understanding. However, few community interventions use critical health literacy and collaborative learning to guide their development and evaluation. Moreover, improving health literacy capacity needs to be seen as a mutual learning process and a shared responsibility between information providers (e.g., professionals) and information users (e.g., patients, citizens). Related to building community-based critical health literacy capacity, this study aims to explore: (1) the needs, assets, and expectations from the perspective of key stakeholders; (2) how collaborative learning influences critical health literacy capacity; (3) what is required to sustain and scale-up such interventions. **Methods:** This study uses a community-based participatory research approach with a qualitative multiple case study design. The study investigates three collaborative learning groups (i.e., cases), which include patients, citizens, community workers, health and social service professionals, librarians, and researchers. Data collection will involve focus group discussions and structured learning journals. Data will be analysed using a hybrid thematic analysis (deductive-inductive). **Results:** Preliminary results will be available in time for the conference (research in progress). **Discussion:** This research addresses the practical need for community-based innovative solutions. The results will be useful to community partners, librarians, health organizations, and educators. Improving critical health literacy will contribute to personal and community care, community development, and health promotion. The project may empower participants to become critical health literacy champions in their communities.

### LT6. Expanded information services during COVID-19: Living background summaries to support drug approval

Kristy Hancock, Leah Boulos
Maritime SPOR SUPPORT Unit

**Introduction:** After an initial surge in potential COVID-19 treatments early in the pandemic, the ever-changing virus and its variants have rendered some treatments less effective and even completely ineffective. Health system decision makers in our province have had to adapt their drug approval process to account for this constantly shifting evidence landscape. As health science librarians, we too have adapted our processes to provide expanded services for these decision makers**. Description:** To support the Nova Scotia Emerging and Re-emerging Infections Therapeutics and Prophylactics Recommendation Group, a group of health system decision makers that advise on approvals for COVID-19 drugs, our evidence synthesis team at the Maritime SPOR SUPPORT Unit maintains a series of living background summaries on COVID-19 treatments. Tailored to the needs of the recommendation group, the summaries compile published and unpublished research, as well as press releases from drug companies, drug approvals in other jurisdictions, unpublished clinical trial results, and news items in an easily digestible format. Summaries are updated on a rotating basis**. Outcomes:** The summaries enable the recommendation group to quickly adapt provincial treatment guidance and develop clinical practice resources using the most up-to-date evidence. Since January 2021, our team has completed over 200

summary updates on 19 different treatments for COVID-19. **Discussion:** The recommendation group's continuous reliance on this service demonstrates the critical role that health science librarians can play in the actualization of evidence. It has led our team to move beyond searching and into packaging contextual information and scholarly research for our users.

### LT7. The Canadian Perspective on the Professional Development Dilemma: A Pilot Study

Margaret Hoogland[1], Angela Spencer[2], Tara Brigham[3]
[1]University of Toledo; [2]Saint Louis University; [3]Mayo Clinic-Florida

**Introduction:** Our study defines professional development as attending webinars, taking a continuing education course, participating in specialized training, attending a conference, presenting (e.g., lightning talk or poster), or publishing an article, book, or book chapter. Our goal was to uncover hidden or less acknowledged reasons (e.g., access to collaborative software, minimal support, protected time) for not completing professional development activities. **Methods:** Our team obtained approval to survey and interview Medical Library Association (MLA) members. After updating the questions, they invited Canadian Health Library Association (CHLA) members to participate in the study. Both CHLA and MLA members completed survey questions on funding for professional development activities, access to collaborative tools, professional development interests, plans for attending conference(s), or completing specialized training in 2023. Interviews with counterpart MLA members asked about attendance and participation at conferences, employer sponsored professional development, ideas for spending grant money, and solicited suggestions for improvement. **Results:** Between February 1 - March 17, 2023; 25 CHLA members completed survey, which closes on March 31st. To date, no CHLA members have scheduled online interviews. Fewer CHLA members (12/25) are required to complete professional development activities compared to MLA members (113/291). Most participants (20/23 CHLA; 230/253 MLA) received some professional development funding. Participants primarily attend conferences to enhance existing skills (13/25 CHLA; 111/248 MLA) or to develop new skills (5/25 CHLA; 133/248 MLA). Participants used funds for national or international conference registration (10/25 CHLA; 93/556 MLA) and membership fees (8/10 CHLA; 67/556 MLA). **Conclusion:** Even with minimal employer support, CHLA and MLA members enjoy completing professional development activities to enhance existing or to develop new skills.

### LT8. Librarian residency - A response to hybrid work

Jennifer McKinnell
McMaster University

**Introduction:** McMaster University requires all units to develop hybrid working arrangements that require bringing employees to campus 2-3 days per week. However, when considering the work of the Health Sciences Librarians, it is almost impossible to define 'campus,' as the librarians work in hospital and campus spaces across the city and the region. The definition of 'day' is also challenging, as the Librarians do not have set hours of work, making it difficult to ask them to work a specific number of hours at any one location. To meet University requirements within this context, the Director of the Health Sciences Library proposed and received approval for a pilot 'Librarian Residency Program.' **Methods:** The Program requires that all Health Sciences Librarians come into the Health Sciences Library, located on the McMaster University campus in Hamilton, Ontario, for three pre-assigned weeks each year. Their remaining time is self-determined. The weeks are meant to foster community building among library employees and build connections with the University. Librarians engage in team-building activities, social events, and planning meetings during residency weeks. They also complete reflection activities. Ongoing program evaluation is required. **Results:** Residency weeks occurred in September 2022, January 2023, and May 2023 and several adjustments have been made. General support for the residency weeks continues, and the Library Director will seek re-approval for the program with modifications. **Conclusion:** Balancing employee work preferences alongside organizational requirements can be challenging, especially when it appears there are conflicting priorities. Residency weeks may be one way to bridge these gaps.

### LT9. How can hospital libraries incorporate principles of equity and social justice in collection development?

Caleb Nault
University Health Network

**Introduction:** Libraries across North America have a problem: census data reports that between 83 to 89% of the librarians employed in Canada and the United States are white, which clearly illustrates a workforce that inadequately reflects the racial diversity of the populations being served in either country (Chan, 2021). Efforts to combat this disappointing reality are numerous and ongoing: various library professional associations have written actions pertaining to the core principles of inclusion, diversity, equity and accessibility (IDEA) into their professional standards, competencies, and strategic priority documents. Collection development and management is an area of librarianship that requires attention, analyses, and transformation. However, attempts to address matters of equity and social justice in collection development and management generally, and in hospital libraries in particular, are fairly nascent (Brillant et al, 2022). How should hospital and health science libraries go about incorporating principles of equity, diversity, inclusion and social justice in the development of their collections? **Description & Discussion:** The ALA (2019) has outlined six ways that libraries can begin working towards building diverse collections through targeted and intentional selection, acquisition, organization, and evaluation practices. This paper discusses an additional five aspects noted in the literature, including ongoing education and self-awareness, challenging the corporatization of social justice via IDEA rhetoric, employing library staff who deeply care about equity, engaging a variety of diversity audits, and leveraging buying power to influence the publishing and library vendor industries. Through exploring key literature, this presentation seeks to open a dialogue about ways to translate IDEA concepts and information into sustainable solutions for developing and managing collections in hospital and health science libraries.

### LT10. Decolonizing bibliographic citations?

Beata Pach, Lindsay Harker, Hannah Skinner, Allison McArther, Domna Kapetanos, Susan Massarella
Public Health Ontario

**Introduction:** Our Library team-part of a government health agency supporting projects related to Indigenous health-was recently asked for guidance citing oral teachings from Indigenous Elders and Knowledge Keepers. Typically, a reference to oral communications, non-existent in many academic style guides, is presented as a footnote. A re-evaluation of this practice was long overdue, thus we set out to draft a citation template in Vancouver style for inclusion in a reference list. **Description:** We examined Canadian, US and Australian university library websites and found variations on Lorisia MacLeod's APA and MLA citation templates, widely accepted as the standard for Indigenous oral teachings-locating one example in Chicago style, but none in Vancouver (our organizational standard). We consulted other relevant literature and guides, reviewed all findings, and discussed each potential element of the citation before attempting an iteration of our own template. **Outcomes:** We became increasingly aware that our Eurocentric conventions were in need of re-examination, and decolonization. A description of our process, and our draft was eventually shared with our corporate advisor on Indigenous strategy and engagement, who identifies as Métis, and who contributed thoughtful, and insightful feedback. Our research in this area indicates that omitting oral teachings from reference lists reproduces colonial practices of devaluing and excluding Indigenous ways of knowing and its dissemination. **Discussion:** We hope to expand this approach into other areas of referencing, by developing citations for other historically underrepresented and undervalued information and media by Indigenous creators, addressing copyright and publishing rights/identity, and following a similar consultative process.

## LT11. Accrediting Library Education Sessions

Stephanie Sanger
McMaster University

**Introduction:** In the Fall of 2015, a relationship was formalized between the McMaster Health Sciences Library and the McMaster Community and Rural Education Program (Mac-CARE). The intent of this relationship was to provide library support for new faculty. Many faculty members did not have access to evidence-based resources at their respective institutions. **Description:** One initiative to support this group was to create a suite of education sessions on topics related to their work as physicians and educators. Physicians are required to complete continuing education and to submit to their accrediting body. The courses developed are accredited for Mainpro+ and the library is in the process of applying for Royal College Maintenace of Certification (MOC) credits. **Outcomes:** Over the years, the library has accredited seven courses and the process of accreditation will be described in this presentation. Discussion: An overview of the history of the partnership between the library and Mac-CARE, the successes of running these sessions, and challenges of putting together the application will be discussed. Future directions will be shared.

## LT12. Update and re-validation of search filters for LGBTQIA+ populations

Hannah Schilperoort[1], Andy Hickner[2], Margaret Foster[3], Chelsea Misquith[4], Jane Morgan-Daniel[5], Kate Saylor[6], Tracy Shields[7], Francis Toole[8], Robin Parker[8]
[1]University of Southern California; [2]Weill Cornell Medicine; [3]Texas A&M; [4]Brown University; [5]University of Florida; [6]University of Michigan; [7]Naval Medical Center Portsmouth; [8]Dalhousie University

**Objectives:** Update and re-validate previously validated PubMed search filters for LGBTQIA+ populations with new MeSH terms and larger gold standard sets for subpopulations. **Methods:** We have reviewed the previously validated LGBTQIA+ and subpopulation filters and added relevant new MeSH and candidate terms. We will create development and validation sets for each subpopulation by extracting studies from systematic reviews that analyze that subpopulation as a unique population. We aim to develop test sets of at least 100 records for each of the development and validation sets. To ensure that the filter applies to a variety of topics, included studies from a minimum of five reviews will be retrieved for each subpopulation. We will conduct term frequency analysis on the citations in the development sets to identify additional terms for the filter and to identify frequently occurring terms. The resulting sensitive and optimized filters will be tested using the validation sets to report the sensitivity and relative recall. To test precision, the search will be run in PubMed and the first 250 records will be retrieved and screened for relevance. **Results:** We will share the search terms, sensitivity, relative recall, and precision for at least one subpopulation filter. **Discussion:** The results of this study will provide researchers validated and reliable search filters for conducting systematic reviews and other types of evidence syntheses with LGBTQIA+ populations. The use of validated search filters will lead to more comprehensive search results and result in higher quality evidence syntheses.

JOURNAL INFORMATION
==============================
NLM Title Abbreviation: J Can Health Libr Assoc
Journal ID: J Can Health Libr Assoc
NLM Journal ID: 101229936
Journal ID: JCHLA

The Journal of the Canadian Health Libraries Association

EISSN: 1708-6892
Publisher: Canadian Health Libraries Association / Association des bibliotèques de la santé du Canada

ARTICLE INFORMATION
==============================
Subjects: LT = Lightning Talk

LT1. Factoring into impact: Librarian involvement on knowledge synthesis projects and relationship to journal impact factor

Alexander Krista
Hall Katharine
Concordia University

JOURNAL INFORMATION
==============================
NLM Title Abbreviation: J Can Health Libr Assoc
Journal ID: J Can Health Libr Assoc
NLM Journal ID: 101229936
Journal ID: JCHLA

The Journal of the Canadian Health Libraries Association

EISSN: 1708-6892
Publisher: Canadian Health Libraries Association / Association des bibliotèques de la santé du Canada

ARTICLE INFORMATION
==============================
Subjects: LT = Lightning Talk

LT2. Diversity audits in health library collections: a methodological exploration

Askin Nicole
University of Manitoba

JOURNAL INFORMATION
==============================
NLM Title Abbreviation: J Can Health Libr Assoc
Journal ID: J Can Health Libr Assoc
NLM Journal ID: 101229936
Journal ID: JCHLA

The Journal of the Canadian Health Libraries Association

EISSN: 1708-6892
Publisher: Canadian Health Libraries Association / Association des bibliotèques de la santé du Canada

ARTICLE INFORMATION
==============================
Subjects: LT = Lightning Talk

LT3. The challenge of accurately formatted references: Does Medline get it right?

Chung Sunny
Scheinfeld Laurel
Stony Brook University

JOURNAL INFORMATION
==============================
NLM Title Abbreviation: J Can Health Libr Assoc
Journal ID: J Can Health Libr Assoc
NLM Journal ID: 101229936
Journal ID: JCHLA

The Journal of the Canadian Health Libraries Association

EISSN: 1708-6892
Publisher: Canadian Health Libraries Association / Association des bibliotèques de la santé du Canada

ARTICLE INFORMATION
==============================
Subjects: LT = Lightning Talk

LT4. Charting Our Sustainable Future: Librarian Philosophies to Discover Meaning in What We Do

Fuhr Justin
University of Manitoba

JOURNAL INFORMATION
==============================
NLM Title Abbreviation: J Can Health Libr Assoc
Journal ID: J Can Health Libr Assoc
NLM Journal ID: 101229936
Journal ID: JCHLA

The Journal of the Canadian Health Libraries Association

EISSN: 1708-6892
Publisher: Canadian Health Libraries Association / Association des bibliotèques de la santé du Canada

ARTICLE INFORMATION
==============================
Subjects: LT = Lightning Talk

LT5. Building Critical Health Literacy Capacity in Partnership with Communities: Research in Progress

Granikov Vera
Centre de recherche du CHUM

JOURNAL INFORMATION
==============================
NLM Title Abbreviation: J Can Health Libr Assoc
Journal ID: J Can Health Libr Assoc
NLM Journal ID: 101229936
Journal ID: JCHLA

The Journal of the Canadian Health Libraries Association

EISSN: 1708-6892
Publisher: Canadian Health Libraries Association / Association des bibliotèques de la santé du Canada

ARTICLE INFORMATION
==============================
Subjects: LT = Lightning Talk

LT6. Expanded information services during COVID-19: Living background summaries to support drug approval

Hancock Kristy
Boulos Leah
Maritime SPOR SUPPORT Unit

JOURNAL INFORMATION
==============================
NLM Title Abbreviation: J Can Health Libr Assoc
Journal ID: J Can Health Libr Assoc
NLM Journal ID: 101229936
Journal ID: JCHLA

The Journal of the Canadian Health Libraries Association

EISSN: 1708-6892
Publisher: Canadian Health Libraries Association / Association des bibliotèques de la santé du Canada

ARTICLE INFORMATION
==============================
Subjects: LT = Lightning Talk

LT7. The Canadian Perspective on the Professional Development Dilemma: A Pilot Study

Hoogland Margaret 1
Spencer Angela 2
Brigham Tara 3
1 University of Toledo
2 Saint Louis University
3 Mayo Clinic-Florida

JOURNAL INFORMATION
==============================
NLM Title Abbreviation: J Can Health Libr Assoc
Journal ID: J Can Health Libr Assoc
NLM Journal ID: 101229936
Journal ID: JCHLA

The Journal of the Canadian Health Libraries Association

EISSN: 1708-6892
Publisher: Canadian Health Libraries Association / Association des bibliotèques de la santé du Canada

ARTICLE INFORMATION
==============================
Subjects: LT = Lightning Talk

LT8. Librarian residency - A response to hybrid work

McKinnell Jennifer
McMaster University

JOURNAL INFORMATION
==============================
NLM Title Abbreviation: J Can Health Libr Assoc
Journal ID: J Can Health Libr Assoc
NLM Journal ID: 101229936
Journal ID: JCHLA

The Journal of the Canadian Health Libraries Association

EISSN: 1708-6892
Publisher: Canadian Health Libraries Association / Association des bibliotèques de la santé du Canada

ARTICLE INFORMATION
==============================
Subjects: LT = Lightning Talk

LT9. How can hospital libraries incorporate principles of equity and social justice in collection development?

Nault Caleb
University Health Network

JOURNAL INFORMATION
==============================
NLM Title Abbreviation: J Can Health Libr Assoc
Journal ID: J Can Health Libr Assoc
NLM Journal ID: 101229936
Journal ID: JCHLA

The Journal of the Canadian Health Libraries Association

EISSN: 1708-6892
Publisher: Canadian Health Libraries Association / Association des bibliotèques de la santé du Canada

ARTICLE INFORMATION
==============================
Subjects: LT = Lightning Talk

LT10. Decolonizing bibliographic citations?

Pach Beata
Harker Lindsay
Skinner Hannah
McArther Allison
Kapetanos Domna
Massarella Susan
Public Health Ontario

JOURNAL INFORMATION
==============================
NLM Title Abbreviation: J Can Health Libr Assoc
Journal ID: J Can Health Libr Assoc
NLM Journal ID: 101229936
Journal ID: JCHLA

The Journal of the Canadian Health Libraries Association

EISSN: 1708-6892
Publisher: Canadian Health Libraries Association / Association des bibliotèques de la santé du Canada

ARTICLE INFORMATION
==============================
Subjects: LT = Lightning Talk

LT11. Accrediting Library Education Sessions

Sanger Stephanie
McMaster University

JOURNAL INFORMATION
==============================
NLM Title Abbreviation: J Can Health Libr Assoc
Journal ID: J Can Health Libr Assoc
NLM Journal ID: 101229936
Journal ID: JCHLA

The Journal of the Canadian Health Libraries Association

EISSN: 1708-6892
Publisher: Canadian Health Libraries Association / Association des bibliotèques de la santé du Canada

ARTICLE INFORMATION
==============================
Subjects: LT = Lightning Talk

LT12. Update and re-validation of search filters for LGBTQIA+ populations

Schilperoort Hannah 1
Hickner Andy 2
Foster Margaret 3
Misquith Chelsea 4
Morgan-Daniel Jane 5
Saylor Kate 6
Shields Tracy 7
Toole Francis 8
Parker Robin 8
1 University of Southern California
2 Weill Cornell Medicine
3 Texas A&M
4 Brown University
5 University of Florida
6 University of Michigan
7 Naval Medical Center Portsmouth
8 Dalhousie University

